# Investigation into the Sensory Properties of Plant-Based Eggs, as Well as Acceptance, Emotional Response, and Use

**DOI:** 10.3390/foods13101454

**Published:** 2024-05-08

**Authors:** Laura Baxter, Emily Dolan, Kaitlyn Frampton, Erin Richelle, Allison Stright, Christopher Ritchie, Rachael Moss, Matthew B. McSweeney

**Affiliations:** School of Nutrition and Dietetics, Acadia University, Wolfville, NS B4P 2K5, Canada; 160338b@acadiau.ca (L.B.); 161731d@acadiau.ca (E.D.); 0210951f@acadiau.ca (K.F.); 159767r@acadiau.ca (E.R.); 160040s@acadiau.ca (A.S.); 151638r@acadiau.ca (C.R.); 145961m@acadiau.ca (R.M.)

**Keywords:** plant-based food, sensory perception, consumer research, sustainability, check-all-that-apply, emotions

## Abstract

Consumers have become interested in plant-based alternatives to animal-based products. One of the under-studied alternatives is plant-based eggs (PBEs). This research investigated PBEs relative to conventional eggs and tofu scramble—another plant-based alternative. Firstly, participants (n = 93) completed a word association task asking them about PBEs. Participants then evaluated the different food samples using hedonic scales, check-all-that-apply (CATA), and temporal check-all-that-apply (TCATA), as well as identified their emotional response and proposed use for PBEs. Participants were interested in plant-based alternatives, including PBEs, but they were concerned about the sensory properties. When they evaluated the different samples, the flavour and texture of the PBEs were disliked in comparison to the eggs. This result may be due to the beany, bitterness, and off-flavour attributes associated with the PBEs. Participants also associated the PBEs with negative emotions. The liking of tofu scramble was not significantly different from the eggs, and the eggs and tofu scramble were mainly associated with positive emotions. During the TCATA evaluation, the participants focused on the flavour attributes of PBEs, while their evaluation of the eggs was dominated by the textural attributes. Whether following a plant-based diet or not, consumers are interested in PBEs, but the sensory properties of PBEs need to be improved before they are willing to adopt them into their diet. This study is one of the first to evaluate the sensory properties of PBEs, as well as consumers’ emotional response to them and their attitudes about PBEs.

## 1. Introduction

Recently, there has been a consumer shift away from animal-based foods [1,2]. The reasons that have been cited for this shift include having a more sustainable or environmentally friendly diet, promoting animal welfare, nutritional benefits, and ensuring global food security [3,4,5,6]. This has led to more consumers following a plant-based diet [7]. As such, the food industry has begun to produce more plant-based alternatives (PBAs). These PBAs are plant-based products that can be substitutes for many conventional animal-based products, including meat, fish, eggs, cheese, yogurt, and milk. PBAs aim to mimic the sensory properties, functionality, and nutritional components of their animal-based counterparts [8,9].

PBAs face many challenges, including high expense, undesirable sensory properties, limited nutritional benefits, and acceptability among consumers [10,11,12,13]. Consumers think that PBAs are expensive [3,14], therefore, lower prices are needed to increase consumer adoption of PBAs. Plant-based diets are promoted for their health benefits [15], but some PBAs are highly processed [16], and many have nutrient profiles that do not meet consumer needs or are quite different from their conventional animal-based counterparts [17,18]. Most PBAs have still been demonstrated to have a better nutritional profile compared to processed animal-based products [16]. Furthermore, food neophobia, a fear of trying new foods [19], has been found to impact consumers’ willingness to try PBAs [20]. Consumers who are trying to follow a flexitarian, vegetarian, or vegan diet tend to have more positive attitudes towards PBAs than those who consume an omnivorous diet [21,22]. Many studies have investigated the sensory properties of plant-based beverages [23,24], cheese [3,12], yogurt [25,26], ice cream, or frozen desserts [27,28], and meat products [29,30]. However, although plant-based eggs (PBEs) have been increasing in popularity, there has been a lack of research investigating the sensory properties and consumer acceptability of PBEs.

Consumers may choose not to consume eggs due to a variety of reasons [31], including health issues (eggs are a source of cholesterol), allergies, sustainability issues (emissions), and animal welfare concerns [14,32,33]. Plant-based eggs are cholesterol-free, plant-based, and sustainable. Other factors that lead to consumers choosing PBEs include the food safety concerns of conventional eggs (such as salmonella), and that PBEs may be easier to handle and store [34]. However, for PBEs to be accepted by consumers they need to have sensory properties that are well-liked by consumers [35]. Although egg replacers for baking applications are an established food product, this study aimed to evaluate PBEs that would be cooked and served similarly to conventional eggs (i.e., scrambled or in an omelette).

Firstly, consumers’ expectations of PBEs were investigated using a word association task (WA). WA asks consumers to write the first four words that come to mind after being presented a stimulus (in this study, PBEs) and allows researchers to identify what expectations consumers have for the food product [36]. To determine the acceptability and the sensory perception of PBEs, hedonic scales and check-all-that-apply (CATA) were used. Hedonic scales and CATA have been used in many studies to evaluate different food items [37,38,39], including PBAs [3,12,23]. Also, temporal check-all-that-apply (TCATA) was used to create a detailed profile of the PBEs [40] as TCATA has been found to not be overly demanding for consumers [41]. Furthermore, emotional responses to PBEs, as well as consumers proposed uses for PBEs, were investigated. As identified by Jaeger et al. [3] product experience is about more than sensory perception, it is also important to evaluate emotional response and to use characteristics.

As such, the goal of this study was to explore Canadians’ expectations for PBEs, as well as their sensory perception using both static (CATA) and dynamic (TCATA) methods, emotional responses, and proposed uses for PBEs. PBEs were compared to conventional eggs, as well as another plant-based alternative—a tofu scramble.

## 2. Materials and Methods

### 2.1. Samples

Three samples were included in this study (eggs, plant-based eggs (PBEs), and a tofu scramble). Eggs (Sobeys, Mississauga, ON, Canada) were included in the study as past studies investigating plant-based products have identified that they are meant to mimic their conventional counterpart and, therefore, they should be included in the sensory trial [3,13,42]. The plant-based eggs (water, mung bean protein isolate, canola oil, corn starch, baking powder, dehydrated onion, dehydrated garlic, transglutaminase, maltodextrin, carotene, turmeric) were commercially available, and at the time of the study, they were the only suitable PBEs available at the grocery stores in NS, Canada. Other plant-based egg products available were marketed as egg replacers for baking. Lastly, the tofu scramble was included in the study based on a survey administered to consumers following a vegan diet, gauging their interest in different plant-based foods (data unpublished). The participants in the survey indicated that they currently consume tofu scramble as a substitute for eggs. Different recipes from the internet for tofu scramble were reviewed, and the tofu scramble used in the trial was created using 454 g of firm tofu (Loblaws Inc., Toronto, ON, Canada), 28 g of nutritional yeast (Loblaws Inc., Toronto, ON, Canada), 3 g of salt (Windsor, Pont de Glace, QC, Ontario), 1 g of turmeric (Club House, London, ON, Canada), 1 g of garlic powder (Loblaws Inc., Toronto, ON, Canada), and 30 g of oat milk (unflavoured and unsweetened; Earth’s Own, Burnaby, BC, Canada).

Vegetable oil was heated in a stainless-steel frying pan over a medium heat on a stovetop before the tofu was added and mashed using a potato masher. The tofu was cooked for approximately four minutes, then nutritional yeast, salt, turmeric, and garlic powder were added. The tofu mixture was then cooked (while stirring) for an additional five minutes. The pan was removed from the heat and oat milk was added and stirred until combined. The PBEs were made as per the manufacturer’s directions, one cup was added to a stainless-steel frying pan with vegetable oil over a medium heat and cooked until all the liquid was cooked through. Scrambled eggs were prepared by thoroughly mixing six eggs with a fork before pouring them into a stainless-steel frying pan heated with vegetable oil over a medium heat. The eggs were cooked for approximately two minutes [43] and were visually inspected to ensure they were cooked.

Samples were cooked immediately prior to evaluation and 30 g of each sample was placed in 2 oz. sample cups. The samples were blinded with random numerical codes. The participants were given a spoon (approximately 7 mL) to eat the samples with and a glass of distilled water to cleanse their palate.

### 2.2. Participants

The 93 participants were adults residing in Nova Scotia, Canada (62 females, 30 males and one participant who preferred not to identify as either, between 18 and 65 years of age). All participants were regular consumers of eggs (monthly or more frequently) and expressed an interest in trying plant-based foods (14% had tried PBEs previously; 61% identified as meat eaters or omnivores, 13% were flexitarians, 13% were vegetarians, 4% were meat avoiders and 4% were pescatarian). All of the participants were screened to ensure that they were not allergic to any of the ingredients in the samples.

### 2.3. Sensory Procedure

The participants read a consent form and gave informed consent before beginning sample evaluation. The sensory questionnaire was presented using Compusense (Version 24.0.26998, Compusense Inc., Guelph, ON, Canada) software on iPads. The participants were seated in sensory booths within the Centre for the Sensory Research of Food (Acadia University, Wolfville, NS, Canada).

First, participants completed a word association (WA) task [36] and were then asked to provide the first four single words or phrases that they thought of when considering PBEs. The words or phrases could include products, emotions, associations, or thoughts.

Secondly, samples were evaluated for their overall acceptability, as well as the liking of their appearance, flavour, and texture using a fully labelled nine-point hedonic scale (1 *=* Dislike Extremely, 9 *=* Like Extremely). Then, the participants’ sensory perceptions were evaluated using CATA. The chosen terms included in the CATA questionnaire were earthy, beany, woody, fried, cabbage, sulphur, salty, sweet, umami (savoury), bitter, astringent, firm, spongy, hard, soft, off-odour, off-flavour, airy, watery, smooth, gritty, buttery, oily, dry, aftertaste, and sour, based on the pilot evaluations and a literature review [3,43,44,45,46]. The terms focused on the flavour and texture of the samples and were presented as outlined by Ares et al. [47]. The participants then evaluated their emotional response to the samples using the EsSense25 profile [48] in the CATA presentation.

The participants received the samples again (blinded with different numerical codes) and evaluated them using TCATA adapted from Ares et al. [41]. The researchers presented the TCATA method to the participants. The participants were instructed to select all the terms from a list that was applied to describe the sensory properties of the sample at each moment of evaluation. Participants had to select the Start button concurrently with taking their first bite of the sample and begin to evaluate the sample. They were informed that the selected property would fade after eight seconds, and if it was still applicable, that they should re-select it. After their first mouthful, they were instructed to take a second mouthful of the sample and continue to evaluate the sample. Participants were instructed to swallow the samples but were not given instructions for at which moment they should do so. The evaluation period was 75 s, but the participants were informed that they could stop the evaluation before this time point if they were not perceiving any sensation. The sensory properties included in the TCATA were salty, savoury, bitter, and beany (four taste/flavour properties) and gritty, smooth, spongy, and dry (four textural properties) [49] based on the preliminary evaluations completed by research assistants employed in the Centre for the Sensory Research of Food.

Lastly, participants were asked to complete an open-ended comment question asking them to identify when they think they would use the PBEs in everyday life and were asked to identify their diet (omnivore, pescatarian, flexitarian, meat avoider, vegetarian, [vegan was not included as the participants had to consume eggs]). The participants then answered demographic questions.

### 2.4. Statistical Analysis

All statistical analysis was performed in XLSTAT (New York, NY, USA). The results of the WA task were analysed as outlined in de Andrade et al., 2016 [50].

Differences in the mean hedonic values were assessed using an ANOVA and Tukey’s Honestly Significant Difference Test. Means and the standard deviations were calculated for each sample. The frequency of the selection of the terms and the emotions selected in the CATA was tabulated (separately). Contingency tables were created for both the sensory properties and the emotional responses, and a Cochran’s Q test was performed for each term and emotion. A correspondence analysis was performed on each contingency table for the terms and emotions [51]. For each CATA term, a penalty lift analysis was completed to evaluate its impact on liking [51].

The results of the TCATA task were analysed in accordance with the procedure outlined by Castura et al. [52]. Briefly, aggregated results across all participants were presented as line plots and the citation proportion of each attribute was calculated. An analysis of the average proportion was calculated using the procedure by McMahon et al. [53].

The results of the comment question were analysed by the researchers, and recurring themes and beliefs were highlighted. The categorization of the results was completed and the results were discussed among the authors to reach a consensus [54]. Descriptive statistics evaluated the demographic questions.

## 3. Results

Eleven different categories were identified by the researchers (n = 93), based on the participants’ responses during the WA task (Table 1). The most frequently mentioned words were in the sensory properties category (28%), and the participants described the different properties they expected to perceive in the PBEs. The second most frequently mentioned category was negative (15%), which identified that the participants had negative expectations for the PBEs. The negative category was followed by ingredients (8%), vegan/plant-based (8%), health (7%), positive (7%), and alternative (6%). Lastly, the participants identified that they were unfamiliar with the PBEs (5%), expected them to be sustainable (4%) and expensive (3%), and compared them to chicken and conventional eggs (3%).

The mean overall liking for the samples ranged between 4.9 to 6.4 on the nine-point hedonic scale (Table 2). These results indicate that the samples ranged from “dislike slightly” (4) to “like slightly” (6) on the scale. The eggs were liked significantly more than the PBEs (*p* < 0.05), and this trend was also seen in the participants’ liking of the flavour and texture of the samples (*p* < 0.05). The flavour of the tofu sample was also liked significantly more than the PBEs (*p* < 0.05). There were no significant differences in the participants liking of the appearance of the samples (*p* > 0.05).

**Figure 1:**
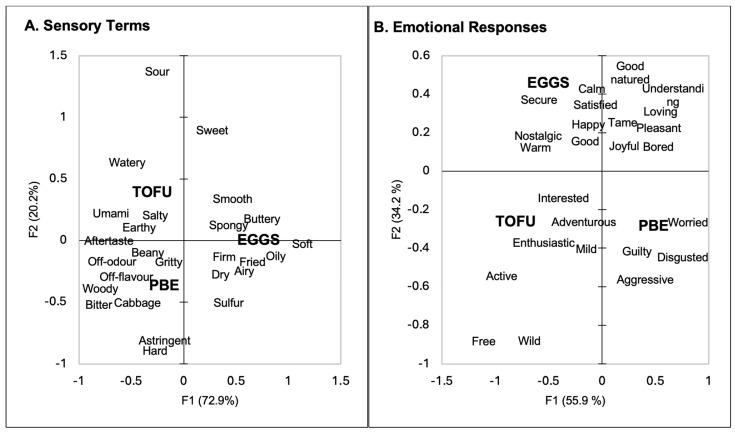
Biplots of the first two dimensions of the Correspondence Analysis of the CATA results for the sensory terms and the emotional responses.

The samples were characterized using both sensory properties (Figure 1A) and emotional responses (Figure 1B). The first two dimensions after Correspondence Analysis can be found in Figure 1. The samples were separated based on their sensory properties (Figure 1A, the first dimension explains 72.9% of the variation, and the second dimension 20.2%), with the eggs on the positive side of the first dimension and the tofu and PBE samples on the negative side. The eggs were associated with the firm, fried, airy, soft, sulphur, buttery, spongy, smooth, and oily attributes and were mainly separated from the other samples based on their textural attributes. The second dimension separated the tofu sample from the PBE sample. The tofu sample was associated with umami, salty, earthy, aftertaste, hard, and watery attributes, while the PBE was associated with beany, gritty, off-odour, off-flavour, woody, bitter, cabbage, and astringent attributes. Overall, the participants separated the three samples.

The emotional association (Figure 1B) separated the samples similarly to the sensory properties. The PBE was mainly associated with negative emotions (guilty, worried, disgusted, and aggressive). The eggs were associated with positive emotions (nostalgia, happy, calm, good-natured). The tofu sample was associated with interested, adventurous, enthusiastic, mild, active, free, and wild attributes.

**Figure 2:**
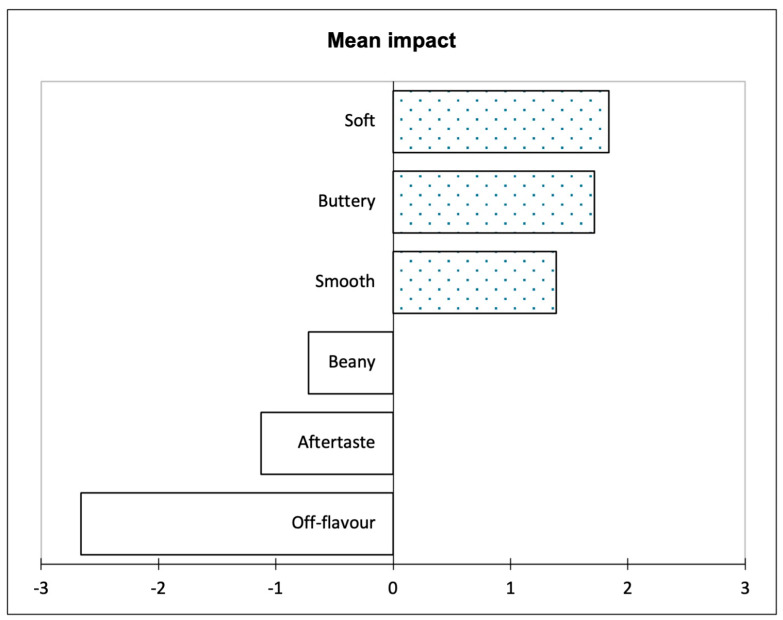
Penalty lift analysis based on the sensory terms and the overall liking of the samples.

The sensory properties were combined with overall liking to identify the drivers of liking (Figure 2). The largest positive impact or hedonic lift was softness, followed by buttery and smoothness. Beany, aftertaste, and off-flavour attributes were found to negatively impact the participants’ liking.

**Figure 3:**
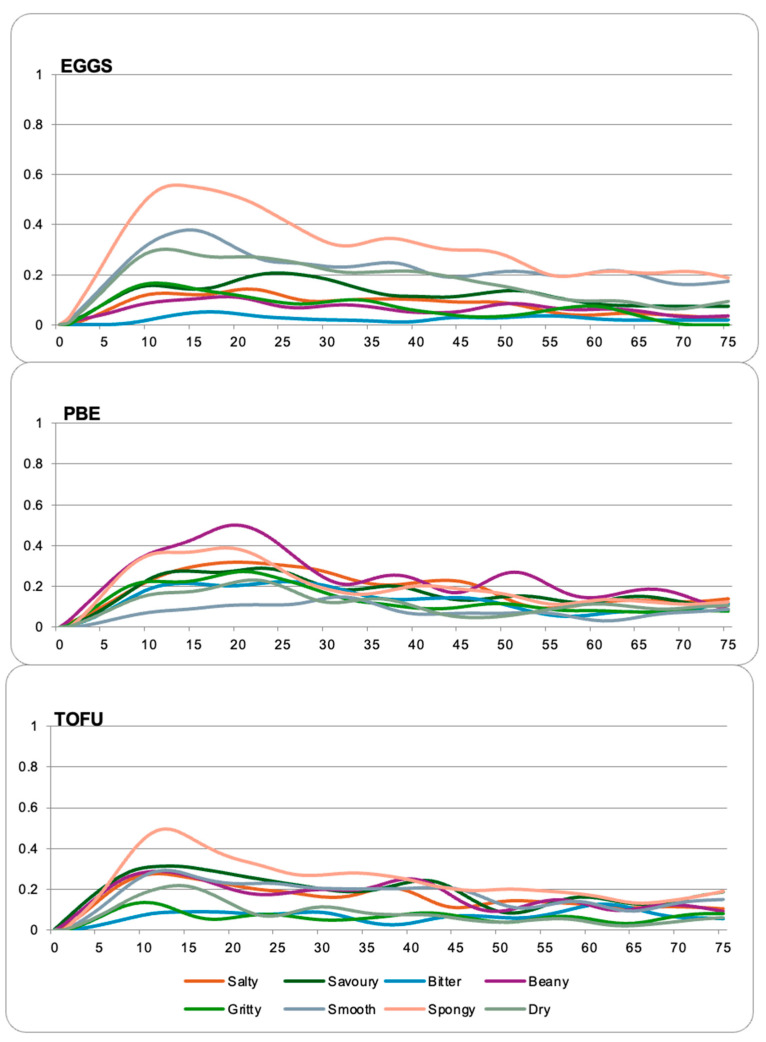
TCATA curves obtained from the participants (n = 93) for the different samples.

Line plots showing the proportion of citations for each sensory property at each time for the different samples are shown in Figure 3. The eggs and tofu were mainly described as spongy, especially in the first twenty seconds of evaluation, while the PBEs were described as beany. The eggs were mainly described using the textural attributes, which were smooth, spongy, and dry. The evaluation of the PBEs was dominated by the taste and flavour attributes (beany, bitter, savoury, and salty) as well as grittiness. The tofu was also found to be salty, beany, and savoury, but was also described as spongy and smooth. The results are reinforced by the average proportions of citations included in Table 3. The PBE was found to be significantly saltier and more beany than other samples, as well as being grittier (*p* < 0.05). The egg samples were significantly less salty, savoury, bitter, and beany than the PBE and tofu samples (*p* < 0.05). Furthermore, on average, smooth, spongy, and dry attributes were selected more often for the egg sample than the tofu and PBE samples (*p* < 0.05).

Lastly, the participants identified how they would use PBEs (Table 4). Participants stated they would use PBEs in cooking and baking applications; however, the participants were concerned about how they would perform in baking applications. Participants also stated they would consume the PBEs daily, while others identified they would only use PBEs if they were cooking for friends or family members who are following a vegan diet or avoiding animal proteins. Many participants (n = 20) identified that they would not use PBEs. Participants were concerned about the price of PBEs and would only use them if they were cheaper or comparable to conventional eggs. Lastly, participants stated that they would use PBEs for their health benefits or if instructed to by their doctor.

## 4. Discussion

The most frequently mentioned category in the WA task was sensory properties. Sensory properties of plant-based alternatives (PBAs) have been found to be important to consumers’ liking of PBAs [11,55]. The sensory properties of PBAs identified by the participants would be considered to negatively impact consumer liking [13,25,56] and agree with the second most mentioned category, negative. This result agrees with a past study which found that consumers may have a negative opinion of PBAs [57]. Studies have also identified that consumers expect PBAs to taste like their conventional counterparts (e.g., milk compared to plant-based beverages). However, if it does not mimic its conventional counterpart, it can lead to hedonic disconfirmation and the disliking of the food item [42,58,59]. If participants had a past negative experience with PBAs, this may have impacted their conceptualization of PBEs. The participants also identified the ingredients they expect to be in PBEs, including soy, nutritional yeast, and coconut oil [10,60]. These ingredients were also included in the samples in this study. This result may indicate that the participants have some familiarity with PBAs, as these are common ingredients.

Participants identified that they associated the PBEs with a plant-based diet and healthiness. In past studies, consumers have been found to believe that plant-based diets and foods are healthy [61,62,63]. Participants in this study had positive responses, indicating that they were interested in PBEs. Participants also identified that the PBEs were an alternative food, as well as listed chicken and egg, reinforcing that consumers do compare PBEs to conventional counterparts. They also stated that the PBEs were sustainable and expensive; these associations have also been found in past studies [64,65]. Furthermore, these results were identified when residents in Nova Scotia, Canada were asked to evaluate plant-based beverages [24]. Lastly, the participants identified they were unfamiliar with PBEs, as would be expected, with only 14% of participants having consumed PBEs previously.

The eggs were liked significantly more than the PBEs in terms of their flavour, texture, and overall liking, but did not differ in terms of liking of appearance. The flavour of the tofu sample was also liked significantly more than the flavour of the PBEs. This result has been found in past studies comparing PBAs to their conventional counterparts [25,27,42]. However, as identified by Cardello et al. [42], there are consumer segments that are plant-based likers who prefer PBAs. The population in this study was not large enough to allow for segmentation, but future studies should investigate if this segment exists for PBEs. This result may be due to the participants’ familiarity with the eggs sample, as familiarity has been found to increase consumer liking [66]. However, all participants were interested in the PBEs, and positive responses were found in the WA task. As identified by Jaeger et al. [3], the consumer acceptability of PBAs will be modest or less than conventional counterparts. In their study, they identified that the “PB alternative cream cheese is within reach” of conventional cream cheese as it was less than one hedonic scale point difference. Using this criterion, the participants liking of the PBEs was within reach of the eggs (PBE = “Neither like nor Dislike” compared to eggs = “Like Slightly”) on the nine-point hedonic scale.

The participants separated the three samples based on their sensory properties (Figure 1A), and the eggs were mainly associated with textural attributes. The texture of eggs is important to consumers and impacts liking [67,68]. The eggs were not associated with many flavour attributes. This result could be because no seasoning was added to the eggs, but seasoning was included in the commercial formulation of the PBEs. Future studies may want to investigate the addition of different seasonings to eggs and their impact on liking when compared to PBEs and tofu. However, the purpose of this study was solely to establish a baseline comparison (eggs to PBEs). The tofu sample was associated with beany, umami, earthy, salty, watery, and aftertaste attributes. It is well established that tofu has a beany flavour [69], and the umami taste can be attributed to the use of nutritional yeast in the tofu scramble recipe [70]. Earthy and aftertaste attributes have been associated with products made from soy [69,71]. The PBEs were associated with off-odour and off-flavour, as well as woody, bitterness, cabbage, astringency, and gritty attributes. Many PBAs have been associated with bitterness and astringency [72,73]. Mung bean isolates have been found to have an intense odour and taste [74] which may explain why consumers associated the PBEs with off-odour and off-flavour. A cabbage off-flavour has additionally been found in other protein isolates (although usually whey protein) [75]. However, the selection of cabbage may also be due to unfamiliar flavours in the PBEs. Overall, the eggs were characterized by their textural attributes, and the PBE and tofu samples were mainly characterized by their flavour attributes.

This result is reinforced by the results of the TCATA (Figure 3 and Table 4), as the eggs had a higher proportion of citations for the textural attributes smooth, spongy, and dry compared to the other samples. Meanwhile, the citation frequencies for all flavours and tastes (salty, savoury, bitter and beany) were significantly higher for the tofu and PBE samples. This result may be due to the different seasonings used in the formulations, but also agrees with the results of CATA as beany, bitterness, umami, and salty attributes were all found on the opposite side of the biplot from the eggs. This result may also be due to familiarity. If the participants were unfamiliar with the flavours of the PBE and tofu samples, they may have focused more on the flavours. In a past study on tropical fruits, an unfamiliarity with the flavours led to negative disconfirmation [76], indicating that unfamiliar flavours impact the consumer perception of foods.

The penalty lift analysis identified that soft, smooth, and buttery attributes all increased the participants’ liking of the samples. All of these attributes were associated with the eggs sample and have been identified in scrambled eggs in past studies [67,68,77]. These are the attributes the participants expect from eggs. Beany, aftertaste, and off-flavour attributes were all found to lead to a disliking of the PBE and tofu samples. Beany and aftertaste attributes have been found in past studies on soybeans and mung beans [71,78,79,80], and as described, the off-flavour may be due to the mung bean isolate in PBEs. Past studies have also identified that the beany flavour, off-flavours, and aftertaste attributes have decreased participants’ liking of PBAs [24,63,81,82]. Overall, the texture of the eggs seemed to increase the participants’ liking, while the flavour of the PBE and tofu sample detracted from their liking.

Many sensory researchers have advocated for research to go “beyond liking” [3,83,84], and as such, this study asked consumers about their emotional responses and potential uses. The eggs were associated with positive emotions and agree with past studies which identified that consumer liking is linked with positive emotions [85,86], as the eggs were the most liked sample. The tofu was also associated with positive emotions, such as adventurous, interested, and enthusiastic. Worried and disgust were associated with PBEs in this study. This result has also been found in past studies where interested consumers evaluated PBAs [11,24]. This result may indicate that PBAs do not currently meet the expectations of interested consumers, which could lead to a negative emotional response. As identified by Piqueras-Fiszman and Spence [59], negativity occurs when a person evaluates a product and their expectations of the product are not met.

Lastly, the participants were asked when they would use PBEs in everyday life, and five categories of responses were identified. One category identified they would not use PBEs, and another stated they would only make those for friends and family members who were following a plant-based diet. This result would indicate that, although they were interested in PBEs, they do not plan to eat them in the future. Other participants stated they would eat them if they were priced cheaper than conventional eggs. Price has been found to impact consumers’ purchase intent of PBAs [87,88], agreeing with this study. Participants also identified that they would be willing to use PBEs in cooking and baking if they functioned similarly to eggs, indicating, similar to other studies, that they expect PBAs to be comparable to their conventional counterparts [89]. The last category of participants said they would consume PBEs daily and the majority of these participants had consumed PBEs previously.

All studies have limitations that need to be addressed. Firstly, this study only included Atlantic Canadians and 14% of participants had previously consumed PBEs; future studies may want to explore the perception of PBEs of those living in other countries or those following a vegan diet. However, this may prevent the comparison of PBEs to eggs if the consumers are following a vegan diet. All of the participants were interested in the PBAs and PBEs and the plant-based foods market is not only targeted at those following a vegan diet, but also hopes to accommodate the needs of consumers hoping to reduce their animal food consumption [89]. Other countries and regions may have different varieties and formulations of PBEs that need to be further explored. Future studies should evaluate the use of the PBEs in different applications to evaluate their functionality and resulting sensory properties. This study focused on the flavour and texture attributes of the PBEs, future studies should evaluate the appearance as well.

## 5. Conclusions

This study evaluated Canadian consumers’ perception of PBEs compared to eggs and another plant-based alternative to eggs—tofu scramble. It is one of the first studies to evaluate the sensory properties, emotional responses, and opinions of PBEs. Participants identified that they were not very familiar with PBEs, had a negative opinion of them, and were worried about the sensory properties of PBEs. The participants’ overall liking, as well as the liking of the flavour and the texture, was significantly less than the eggs. This result may be due to the beany, bitterness, and off-flavour attributes associated with the PBEs. The PBEs were also associated with negative emotions (disgusted and worried). There was no difference in the liking of the appearance of the PBEs when compared to the eggs, and the PBEs were found to be saltier and more savoury than the eggs during the TCATA task. The PBEs were not well-liked by the consumers when compared to the eggs and tofu scramble, and many consumers did not see themselves eating them in the future (or only cooking them for those who do not consume eggs). PBEs face many challenges, as identified by the participants, including price and undesirable sensory properties, but there is potential for them as participants identified they are sustainable and have health benefits. Also, a subsection of the participants identified they could eat the PBEs every day and more participants may have had this response if all participants were familiar with PBEs. Future research should be conducted investigating how PBEs compare to eggs when used in different cooking applications and investigate PBEs made from different ingredients. Further research investigating the impact of familiarity on consumer perception should be conducted. Also, the environmental consciousness of the consumers and its’ influence on consumer perception of PBEs should be evaluated.

## Figures and Tables

**Table 1:** Frequency of the mention of the categories and examples of individual associations from the participants during the Word Association Task.

Categories	Examples	Percentage of Mention
Sensory Properties	Rubbery, weird texture, tasty, off-flavour, bland, watery, no flavour	28%
Negative	Yuck, ew, unappealing, weird, gross, strange, is it safe to eat?	15%
Ingredients	Soy, tofu, nutritional yeast, coconut oil, salt, soybeans	8%
Vegan/Plant-based	Vegan, vegan diets, plant-based, plant-based diets, plants	8%
Health	Healthy, low cholesterol, protein source, nutritious, iron	7%
Positive	Good, interesting, potential, finally!, curious, cool	7%
Alternative	Substitute, alternative, different, breakfast alternative, fake	6%
Unfamiliar	Confused, unfamiliar, strange, unfamiliar, not aware	5%
Sustainable	Sustainable, green, plant-based, environmental, environmentally friendly	4%
Expensive	Expensive, price, costs a lot, money	3%
Chicken/Eggs	Chicken, eggs	3%
Uncategorized	Natural, not sure, organic, lab grown, cows, huh?	6%

**Table 2:** The mean liking scores (1 = Dislike Extremely and 9 = Like Extremely) of the samples (eggs, PBE and tofu scramble) included in the study with the standard deviation in brackets (n = 93). Within columns, the samples with different letters had significantly different means at the 5% level of significance.

Sample	Appearance	Flavour	Texture	Overall Liking
Eggs	5.6 a (1.3)	6.6 a (1.1)	6.1 a (1.2)	6.4 a (1.3)
PBE	5.9 a (1.8)	4.7 b (1.1)	5.0 b (1.7)	4.9 b (1.5)
Tofu	5.3 a (1.8)	5.9 a (1.6)	5.7 ab (1.7)	5.6 ab (1.6)

**Table 3:** The average proportion of citations of the sensory terms during the TCATA task for each sample (n = 93). Means in the same row, with the same letter, are not significantly different at α = 0.05.

Attribute	Eggs	PBE	Tofu
Salty	0.080 c	0.190 a	0.160 b
Savoury	0.120 b	0.171 a	0.192 a
Bitter	0.022 c	0.129 a	0.068 b
Beany	0.065 c	0.248 a	0.169 b
Gritty	0.072 b	0.134 a	0.064 b
Smooth	0.222 a	0.076 c	0.172 b
Spongy	0.312 a	0.198 b	0.242 b
Dry	0.173 a	0.115 b	0.080 c

**Table 4:** Results of the comment analysis pertaining to the proposed use of the plant-based eggs.

Category	Summary of Response Identified
Cooking	Scrambled, casserole, breakfast burritos, breakfast sandwich, baking, baked goods, noodle dishes, in fried rice, breakfast wraps, would bake with them if like eggs, scrambled with cheese.
Daily	Daily, every day if they were quality.
Plant-based	Vegan diet, cooking for a vegan person, when avoiding animal proteins, cooking for vegetarian or vegan friends.
Negative	No other options, I would not, only if I had to, never, I would not, not often.
Price	If cheaper, if comparable in price to regular eggs, if affordable, if cheaper than normal eggs.
Health	If healthier I would, if recommended tom by my doctor for health, to avoid unhealthy steroids and pesticides, to improve my diet.

## Data Availability

The original contributions presented in the study are included in the article, further inquiries can be directed to the corresponding author.
